# Mesoporous Silica-gold Films for Straightforward, Highly Reproducible Monitoring of Mercury Traces in Water

**DOI:** 10.3390/nano9010035

**Published:** 2018-12-28

**Authors:** Anna Mutschler, Vivian Stock, Lena Ebert, Emma M. Björk, Kerstin Leopold, Mika Lindén

**Affiliations:** 1 Department of Inorganic Chemistry II, Ulm University, Albert-Einstein-Allee 11, 89081 Ulm, Germany; anna.mutschler@uni-ulm.de (A.M.); lena.ebert@ac.rwth-aachen.de (L.E.); emma.bjork@liu.se (E.M.B.); 2 Department of Analytical and Bioanalytical Chemistry, Ulm University, Albert-Einstein-Allee 11, 89081 Ulm, Germany; vivian.stock@uni-ulm.de; 3 Department of Physics, Chemistry, and Biology, Linköping University, 58183 Linköping, Sweden

**Keywords:** gold nanoparticles, mesoporous silica films, mercury trace analysis

## Abstract

Trace-level detection of mercury in waters is connected with several complications including complex multistep analysis routines, applying additional, harmful reagents increasing the risk of contamination, and the need for expensive analysis equipment. Here, we present a straightforward reagent-free approach for mercury trace determination using a novel thin film sampling stick for passive sampling based on gold nanoparticles. The nanoparticles supported on a silicon wafer and further covered with a thin layer of mesoporous silica. The mesoporous silica layer is acting as a protection layer preventing gold desorption upon exposure to water. The gold nanoparticles are created by thermal treatment of a homogenous gold layer on silicon wafer prepared by vacuum evaporation. This gold-covered substrate is subsequently covered by a layer of mesoporous silica through dip-coating. Dissolved mercury ions are extracted from a water sample, e.g., river water, by incorporation into the gold matrix in a diffusion-controlled manner. Thus, the amount of mercury accumulated during sampling depends on the mercury concentration of the water sample, the accumulation time, as well as the size of the substrate. Therefore, the experimental conditions can be chosen to fit any given mercury concentration level without loss of sensitivity. Determination of the mercury amount collected on the stick is performed after thermal desorption of mercury in the gas phase using atomic fluorescence spectrometry. Furthermore, the substrates can be re-used several tens of times without any loss of performance, and the batch-to-batch variations are minimal. Therefore, the nanogold-mesoporous silica sampling substrates allow for highly sensitive, simple, and reagent-free determination of mercury trace concentrations in waters, which should also be applicable for on-site analysis. Successful validation of the method was shown by measurement of mercury concentration in the certified reference material ORMS-5, a river water.

## 1. Introduction

The toxicity of mercury and its compounds is well known today and the United Nations Environment Program considers mercury as one of the most critical environmental pollutants. In 2017 the Minamata Convention on Mercury [1], an international treaty to protect human health and the environment from the adverse effects of mercury, was ratified by more than 90 states who have recognized that mercury is “a chemical of global concern owing to its long-range atmospheric transport, its persistence in the environment once anthropogenically introduced, its ability to bio-accumulate in ecosystems and its significant negative effects on human health and the environment ” [1]. Mercury is emitted to the environment mostly in its elemental form (Hg(0)) by both, natural sources, like volcanic eruption, as well as by anthropogenic sources, mainly by combustion of fossil fuels and small scale gold mining. These two sources account for more than 60% of the global anthropogenic mercury emissions and were rising considerably in the last decade [2]. Therefore, even though efforts to reduce mercury emissions by reduction of its usage in industrial processes (like chlorine-alkali electrolysis) and commercial products (e.g., electrical and electronic devices) were successful [3], the global overall anthropogenic mercury emissions rose. Once emitted mercury is globally distributed due to its long lifetime in the atmosphere. Wet and dry deposition lead then to elevated mercury levels in the hydrosphere [4] where extremely high bioaccumulation factors of up to 10^6^ cause considerable contamination of eatable fish. Fish consumption is therefore nowadays the main source for non-occupational human mercury exposure [5,6]. Accordingly, monitoring of mercury traces in the hydrosphere has been regulated in many countries, e.g., in Europe by the European Water Framework Directive [7]. Moreover, the Minamata Convention on Mercury includes beside provisions related to reductions of usage and emission of mercury also regulations regarding controls and monitoring of mercury immission. This implies that many countries in which monitoring of mercury has not yet been established agreed to introduce according analysis programs.

In pristine natural waters, mercury concentrations vary between pg and ng·L^−1^ range, while contaminated waters at hot spots may contain up to several µg·L^−1^. However, reliable quantification of mercury traces in natural waters is challenging due the omnipresence of Hg at low levels. Various highly sensitive and selective analytical methods are available for this purpose. Instrumental techniques like atomic absorption spectrometry (AAS) [8], atomic fluorescence spectrometry (AFS) [9], and inductively coupled plasma - mass spectrometry (ICP-MS) [10] are usually applied. Typically, these techniques are coupled to an efficient separation technique, mostly chemical cold vapor generation (CV). Thereby, addition of reagents transforms mercury species into “reducible mercury” (Hg^2+^). Subsequently chemical reduction to gaseous elemental mercury (Hg(0)) and its separation from the matrix by purging is performed. In addition, to obtain highest sensitivity Hg(0) vapor can be pre-concentrated by the amalgamation technique (AT). Commercially available amalgamation traps are either made from silver- or gold- coated and [8] and silica wool [9] or consist of bulk materials like gold foil strips [10] and Au/Pt gauzes [11]. Coupling CV, AT, and atomic/mass spectrometry provides high sensitivity mercury detection with detection limits in the low pg·L^−1^-range [12]. Nevertheless, there are several disadvantages associated with such an approach, like time-consuming analyses, multi-step sample preparation, use of various additional reagents that result in increased risk of contamination, high blank values and consequently compromise achievable detection limits. Moreover, the elaborate sample preparation and bulky instrumental equipment hampers on-site application and typically transport of water samples from the sampling site into the laboratory is performed. Therefore, novel analysis strategies that overcome these problems are required. Thereby, nanomaterials are considered by several researchers as a tool to enhance analytical procedures in trace metal analysis [13,14]. Suggested approaches for mercury trace analysis include the application of silver nanoparticles [15], oxidized carbon nanotubes [16], imprinted polymeric nanoparticles [17], polymeric nanofibres [18], or magnetic nanoparticles [19,20] for pre-concentration and separation of mercury. In former work we reported on the development of a simplified laboratory procedure using nano-structured gold collectors for reagent-free pre-concentration of Hg species from natural waters [21,22]. In contrast to commercially available gold traps for Hg(0) pre-concentration, these nano-structured gold collectors accumulate also oxidized and alkylated Hg species and so determination of total dissolved Hg is achieved. Thermal desorption of mercury from the collectors allows subsequent detection of released Hg(0) by any selected atomic spectrometric technique. Furthermore, we have recently shown that meso-macroporous silica monoliths loaded with gold nanoparticles are efficient substrates for solid phase extraction of total Hg from water samples, providing a limit of detection as low as 1.31 ng Hg L^−1^ for only one-minute accumulation duration, when coupled to AFS. Despite the excellent performance of the gold-silica monoliths for mercury accumulation, there are several drawbacks related to their practical use as on-site sampling device. The monoliths themselves show a limited mechanical stability, and there are serious issues related to the homogeneity and reproducibility of gold loading into the monoliths. Furthermore, sampling-desorption cycling of the monoliths is limited by the morphological stability of the monoliths, and they can easily crack upon fast drying due to the high capillary pressures arising from the high surface tension of water and the small pores in the silica matrix. Thus, we have extended our investigations towards novel nanogold-mesoporous silica films on mechanically stable silicon wafer substrates for their application as robust and recyclable Hg sampling sticks.

## 2. Materials and Methods 

### 2.1. Chemicals Used

Ethanol (Merck, absolute for analysis, Darmstadt, Germany), Tetrathylorthosilicate (TEOS) (VWR International S.A.S.), Pluronic^®^ F-127 (Sigma-Aldrich Darmstadt, Germany), hydrochloric acid, technical acetone (VWR Chemicals, UN 1090), acetone (Technic France, UN 1090), isopropanol (Technic France, UN 1219), deionized water, ultrapure water. Argon gas (99.996%, MTI Industriegas AG), Hydrochloric acid 37% (p.a. EMSURE^®^, Merck, Darmstadt, Germany), Mercury standard solution (traceable to SRM from NIST, Hg(NO_3_)_2_ in HNO_3_ 2 mol·L^−1^, Merck, Darmstadt, Germany), Vanadium standard solution (traceable to SRM from NIST, NH_4_VO_3_ in HNO_3_ 0.5 mol·L^−1^, CertiPUR^®^, Merck, Darmstadt, Germany), Tin(II) chloride (≤0.000001% Hg, p.a. EMSURE^®^, Merck, Darmstadt, Germany). 

### 2.2. Preparation of Gold nanoparticle-covered Silicon Wafers

Si-wafers (Silicon Materials, P/Bor<100>) were used as substrates. These were cut into 4 × 0.5 cm^2^ pieces and cleaned with acetone in an ultrasonic bath. Before vacuum evaporation of the gold layer on to the substrates theses were cleaned in a warm acetone bath followed by an isopropanol and ultrapure water bath, and finally dried under an ultrapure N_2_ flow. The cleaned and dried substrates were coated with a uniform gold-layer with a thickness of about 6 nm through vacuum evaporation. The gold-coated silicon wafers were heat-treated in air for 2 h at 270 °C on a hotplate, which lead to the formation of gold nanoparticles on the silicon substrates. 

### 2.3. Preparation of the Mesoporous Silica Top-layer

Mesoporous silica films were prepared by dip-coating (Sol-Gel Way, Paris, France) using a method previously described by Faustini et al. [23] and Cagnol et al. [24]. In a typical synthesis, 36.5 mL ethanol, 1.4mL water and 37.6 µL HCl (2 M) were mixed in a 100 mL round-bottomed flask. Under stirring at 350 rpm and RT 3.46 mL TEOS a 1.17 g Pluronic^®^ F-127 were added and stirred until the sol became transparent. The sol was aged for 24 h (350 rpm, RT) before dip-coating. The dipping speed typically used was 2.5 mm·s^−1^ and the dipping was carried out at a relative humidity of about 70%. The films were further aged for 30 min at 70% relative humidity before calcination. Calcination was performed in an oven at a temperature of 400 °C for 5 min (heating rate 20 °C·min^−1^). 

### 2.4. Film Characterization

Scanning electron microscopy (SEM) was performed on a Hitachi S-5200 and Helios Nanolab 600 operated at 10 kV. Cross-section images were obtained with focused ion beam scanning electron microscopy (FIB-SEM) on a Helios Nanolab 600 operated at 5 kV. For the FIB-SEM investigations, a layer of platinum was sputtered onto the area of interest before milling. Transmission electron microscopy (TEM) images of silica films prepared under identical conditions as the gold-silica films but using a removable aluminium foil as the substrate were recorded using a Jeol 1400 setup operated at 120 kV. The dried and calcined film was embedded in an epoxy resin before substrate removal, followed by an additional epoxy resin treatment and cut into thin slices before imaging. The Kr-sorption measurements were performed at −196 °C on a Micromeritics ASAP2020 setup. X-ray diffraction measurements were performed using a Panalytical X’Pert Pro system equipped with an X򲀙Celerator detector and operated in reflection mode. 

### 2.5. Film Stability Investigations and Quantification of Gold Load

Gold stability experiments were performed immersing sampling sticks in 5 mL of a 0.5% (*v*/*v*) HCl solution on an orbital shaker at 230 rpm for 5 min. An aliquot of 4950 µL of this solution was taken and 50 µL of a 100 µg L^−1^ vanadium standard solution were added as internal standard for subsequently investigation of gold content by total reflection X-ray fluorescence spectrometry (TXRF). The sample was thoroughly mixed and 10 µL were applied onto a silicone-coated quartz glass carrier and dried on a heating plate at 70 °C. TXRF measurements were carried out with an S2 Picofox (Bruker AXS GmbH) operating at a tube voltage of 50 kV, a current rating of 600 µA and a lifetime of 1000 s. The limit of detection for Au was 0.17 µg·L^−1^. 

Gold load of a representative sampling stick was measured after extraction of gold in 5 mL *aqua regia*. This was repeated once in order to check if extraction of gold was quantitative. Then, 150 µL of each sample (1st and 2nd extraction) were diluted in 9.85 mL of a 0.5% (*v*/*v*) HCl aqueous solution, mixed thoroughly and measured by atomic absorption spectrometry (AAS) with a ContrAA 600 (Analytik Jena AG, Jena, Germany) equipped with a graphite furnace atomization unit and a liquid dosing using the most sensitive line of Au (242.795 nm). Calibration was performed in a concentration range from of 20 to 70 µg Au L^−1^ providing a detection limit of 3.2 µg Au L^−1^. Gold measurement in the 2nd extraction was below LOD, revealing quantitative extraction in the 1st step.

### 2.6. Mercury Accumulation and Investigation of Analytical Performance for Hg Quantification

**Figure 1:**
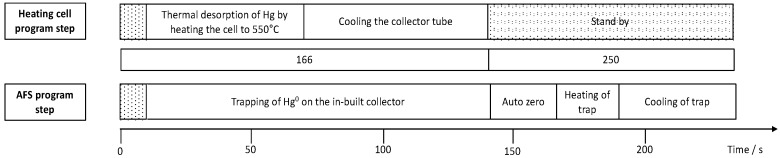
Sequence of program steps for the collector tube for thermal desorption of mercury from the sampling stick coupled to atomic fluorescence spectrometry (AFS) for quantification of accumulated mercury.

Mercury accumulation experiments were performed in 6 mL of a Hg^2+^ containing solution at room temperature on an orbital shaker at 230 rpm. For concentration-dependent measurements, an accumulation time of 5 min and concentrations from 5 to 25 ng Hg^2+^ L^−1^ were chosen. For time-dependent measurements a concentration of 100 ng Hg^2+^ L^−1^ was selected and exposure times were varied from 10 to 390 s. After accumulation, the sampling sticks were rinsed with UPW and placed in a collector tube coupled to an atomic fluorescence spectrometer (AFS, Mercur, Analytik Jena AG, Jena, Germany) as described first by Huber et al. [19]. Figure 1 shows the timelines and parameters for mercury release from the sampling sticks in the collector tube and the AFS instrumentation for mercury detection. An argon flow transports the released Hg(0) to an in-built collector of the Mercur instrument, where it is pre-concentrated and then again released to be transported to the measurement flow-through cell of the instrument. AFS measurements were performed at a wavelength of 253.7 nm and a detection voltage of 391 V. In order to be able to determine masses of Hg released from the sticks a calibration of the instrument was performed using the cold vapor technique. For the online generation of Hg(0), a carrier solution of 0.5% (*v*/*v*) HCl and a reducing solution of a 1.25% (*v*/*v*) HCl containing 0.65% (*w*/*v*) SnCl_2_ were used. Hg(II) standard solutions were prepared freshly by adequate dilution of a stock standard. The sample volume used for the calibration was 2.2 mL resulting in the calibration function y = 0.0017 L·ng^−1^ × −8 × 10^−5^ with R^2^ = 0.9992 and a limit of detection of 0.8 ng Hg L^−1^. The corresponding calibration function is shown in the supporting information in Figure S1.

Validity of the proposed method was checked by measurement of certified reference material ORMS-5: Elevated Mercury in River Water, which was purchased from the National Research Council Canada (NRC, Canada) and handled according to the recommendations given in the certificate. For this purpose, two individual sampling sticks were calibrated in a concentration range from 15 to 35 ng Hg L^−1^ using Hg(II) standard solutions and then applied to 6 mL of the CRM sample allowing an accumulation time of 5 min. Accumulation, thermal desorption and subsequent AFS measurement were performed 4-times with each stick (*n* = 4).

## 3. Results and Discussion

### 3.1. Synthesis and Characterization of Gold Nanoparticle-coated Substrates

**Figure 2:**
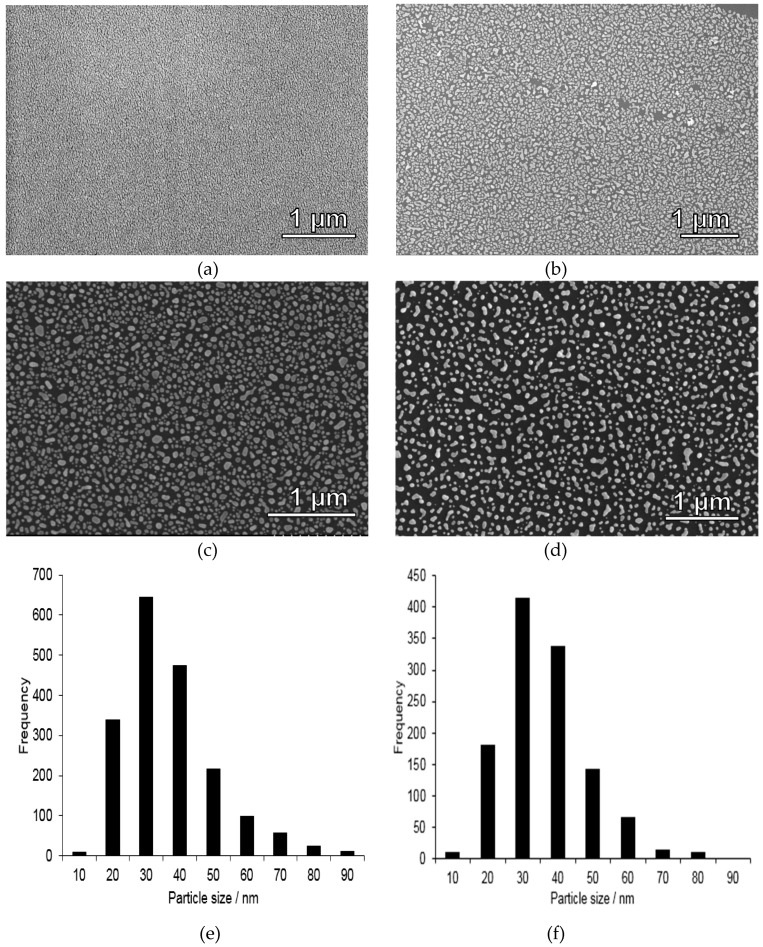
SEM images taken of gold-coated silicon substrates (**a**) before thermal treatment, (**b**) after thermal treatment at 270 °C for 2 h in air on a hot plate, (**c**) after calcination for 5 min at 400 °C, (**d**) same heat treatment as substrate (**c**) but with additional washing steps afterwards, showing that some gold nanoparticles detach from the substrate during washing step, and thus such films are not suitable as such for mercury accumulation, and (**e**) corresponding gold particle size histogram for (**c**) and (**f**) size histogram for (**d**), showing that the gold nanoparticle size distribution was not affected by the further heat treatment at 400 °C. As the particles were irregular in shape, the shortest lateral cross-section distance is shown in the histogram.

Silicon wafers were chosen as substrates due to their mechanical stability, flatness, and chemical durability. Gold nanoparticle-coated substrates were prepared through thermal treatment of a homogeneous, 6 nm thin layer of gold vacuum evaporated onto a silicon wafer. Thermal dewetting of the substrate leads to the formation of relatively evenly spaced islands of gold. With increasing the gold film thickness the dewetting process decelerates, greater islands are formed and the spacing increases [25]. Although other means for depositing gold nanoparticles are available, for example, pulsed laser technology [26], the thermal dewetting approach was used in our studies due to its straightforwardness and easy scalability. SEM images of gold-coated substrates before and after thermal treatment at 270 °C for 2 h on a hot-plate are shown in Figure 2a,b. Also included is a histogram of the gold nanoparticle diameters after this treatment step, Figure 2f. Irregularly shaped gold nanoparticles exhibiting a relatively broad particle size distribution ranging from about 20–90 nm, and which was peaking at 30 ± 5 nm. The films were further heat-treated at 400 °C for 5 min, and the corresponding SEM image is shown in Figure 2c, and the histogram in Figure 2e. As can be seen, the particle size distribution was hardly affected by this additional heat-treatment step. Similar gold nanoparticle sizes have previously been observed for 5 nm thick homogeneous gold layer deposited onto a glass substrate and which subsequently were heat-treated at 550 °C for 10 h [27], and for a 5 nm thick gold layer deposited on a silicon substrate followed by annealing at 950 °C for 10 min in air [28]. In order to check the adhesion of the nanoparticles to the silicon substrate, the calcined film was further subjected to a washing step identical to that used for sample preparation before mercury analysis (see experimental). The corresponding SEM image is shown in Figure 2d. As can be seen, part of the gold particles are detaching upon washing, showing that “naked” gold nanoparticle films as those studied here cannot be applied for quantitative mercury analysis, as discussed in more detail below.

**Figure 3:**
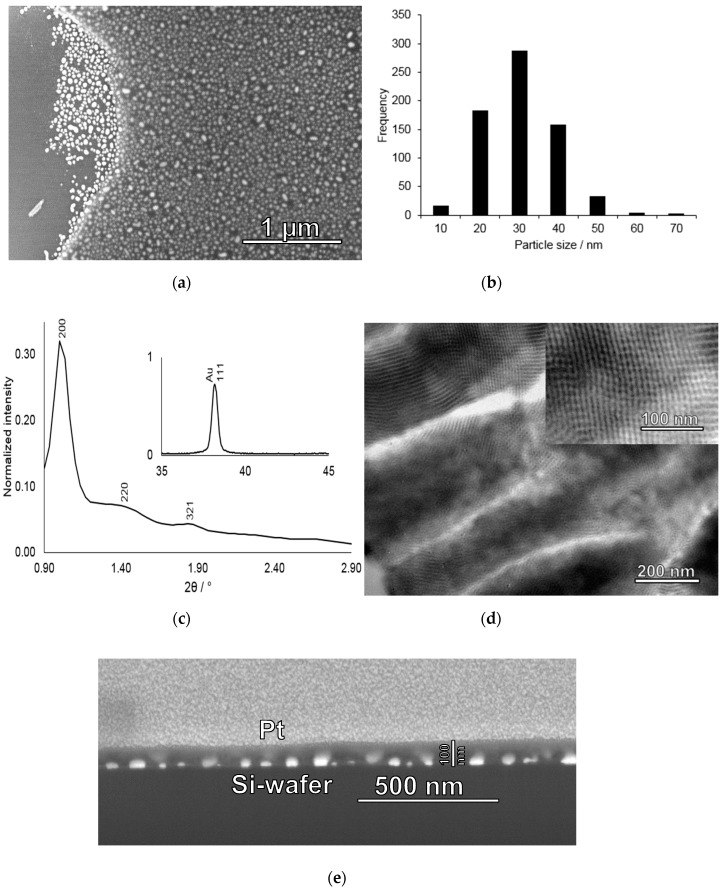
Characterization of gold nanoparticle-mesoporous silica film deposited on a silicon wafer. (**a**) SEM image of a dip-stick where the silica film had partly detached from the silicon substrate and (**b**) corresponding histogram of the gold nanoparticle sizes determined from the silica-film-free area in a); (**c**) Low-angle X-ray diffraction pattern measured for a calcined gold nanoparticle-mesoporous silica film showing three identifiable reflexes in the low-angle region which can be indexed as the 200, 220, and 321 reflexes characteristic of the Im-3m space group. Inset: Wide-angle region showing the 111 reflection of the gold nanoparticles, (**d**) Cross-section TEM image taken of a mesoporous silica film deposited on an aluminium foil and prepared under identical conditions as those used during preparation of the gold-mesoporous silica films on silicon wafers. Inset: Zoom showing the cubic mesostructure of the film; (**e**) FIB-SEM cross-section image of a gold nanoparticle-mesoporous silica film deposited onto a silicon wafer. The area of interest was covered by a layer of platinum through sputtering before milling.

Gold nanoparticle coated substrates without pre-washing were coated with a layer of mesostructured silica using dip-coating. TEOS was used as the silica-source and F-127 as the structure-directing agent, following a procedure published by Faustini et al. [23]. After the dip-coating process, the films were dried followed by calcination at 400 °C in order to remove the surfactant and to open up the pores. An SEM image taken of a film where the mesoporous silica film had partly detached from the silicon substrate during sample preparation is shown in Figure 3a. The gold nanoparticles are homogeneously coated by the silica film and show a high level of homogeneity, showing that the gold nanoparticles did not detach during film formation or upon thermal processing. The gold particle size distribution profile was similar to those observed for the gold-only films, but was slightly shifted towards smaller particle sizes as compared to the naked gold nanoparticle films. However, also here the particle size distribution peaks at 30 ± 5 nm. The size reduction is suggested to be due to some gold evaporation during the thermal treatment step for the mesoporous silica covered gold nanoparticles. The decrease in the gold nanoparticle size upon thermal treatment together with the observation that the gold nanoparticles are firmly attached to the substrate suggests that there is empty space between the gold nanoparticles and the silica film. This is important, as the incorporation of mercury into the gold nanoparticles (amalgamation) leads to a volume increase of the particles, and which otherwise could induce crack formation in the silica film. 

The XRD patterns measured after calcination shown in Figure 3c exhibits several low-angle reflections giving evidence for long-range mesoscopic order in the silica film, and which can be indexed assuming an *Im-3m* space group (body-centered cubic). The calculated unit cell size was 17.2 nm, in good agreement with the literature. Furthermore, as shown in the inset in Figure 3c, high-angle reflections of the gold particles are also present. Based on the FWHM of the 111 reflection at 38.1^o^ 2Θ, a mean crystallite size of 29 nm was calculated using a K-value of 0.9, in good agreement with the particle size evaluation based on the SEM (Figure 3b). TEM images of a silica film deposited under identical conditions but using removable alumina foil as the substrate (see experimental for details) without gold particles is shown in Figure 3d, giving further evidence for a homogeneous porosity and a cubic structure of the mesoporous silica film. Furthermore, estimations of the mesopore diameter based on the TEM image suggest a pore size of about 6–7 nm. The mean film thickness as determined by studying film cross-sections by FIB-SEM was about 100 nm, as shown in Figure 3e. This film thickness was chosen in order to ensure that all gold particles indeed were covered by silica and that the films were crack-free, still keeping the film thickness small in order to minimize potential diffusion limitations upon mercury enrichment. Interestingly, indications for gold evaporation is also seen in the FIB-SEM cross-section image shown in Figure 3e, where areas of higher electron densities can be seen in the silica film on top of the gold nanoparticles probably corresponding to evaporated gold that has re-condensed onto the mesoporous silica.

The BET surface area of the films were measured by Krypton sorption measurements performed at −196 °C. The films had a surface area of about 1000 m^2^·cm^−3^ (0.01 m^2^·cm^−2^), which is in a good agreement with the expected values for a homogeneously mesoporous silica film prepared as described.

In order to determine the gold nanoparticle size distribution after calcination of the mesoporous silica-coated particulate gold films, we studied an area of the film where part of the silica film had been detached upon sample preparation by SEM. The strong contrast difference between the gold nanoparticles covered by the silica film and those not covered by the film, gives strong support for the conclusion made above based on the FIB-SEM results that all gold nanoparticles are indeed homogeneously covered by the mesoporous silica film. The corresponding image is shown in Figure 3. Gold nanoparticle size determination based on image analysis rendered a mean size of 26 ± 9 nm with a size distribution ranging from 6 nm to 63 nm. 

Further support for the importance of a homogeneous mesoporous silica film for not losing gold upon exposing the films to water is that 259.4 ng of gold was lost already during two short washing cycles from a 1.5 cm^2^ nanoparticulate gold film (see Figure 2d), and detectable amounts of gold were also lost during washing of films where the mesoporous silica layer contained cracks as often was the case for thicker mesoporous silica films. 

The amount of gold lost during washing of gold-mesoporous silica films where the silica film exhibited no cracks was below the limit of detection of 1.0 ng Au even after 4 washing cycles, suggesting that also locking in of gold nanoparticles by the mesoporous silica film is important for application of these films in aqueous environment. Furthermore, these results also corroborate the conclusion made above that the gold nanoparticles indeed were homogeneously covered by a mesoporous silica film in these cases. Measurement of gold amount of a representative ready made sampling stick by extracting the gold in *aqua regia* further confirms the effectiveness of the protective layer since 10.26 ± 0.17 µg Au/cm^2^ (mean ± 1SD, *n* = 3) were found, which is close to the original, nominal mass when depositing a 6 nm thin film (approx. 11µg Au/cm^2^).

### 3.2. Mercury Accumulation on the Gold-Mesoporous Silica Films

**Figure 4:**
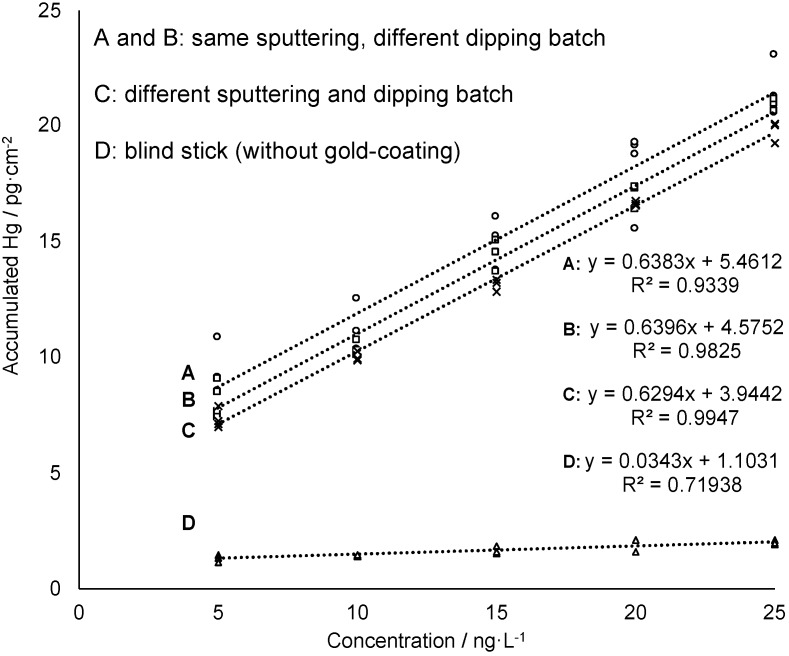
Concentration-dependent measurements of sampling sticks from different sputtering and/or dipping batches. The sampling sticks for the calibration A and B are from the same sputtering but different dipping batches, C is from a different sputtering and dipping batch and D is the blind stick. The accumulation test was performed in 6 mL sample solution with an accumulation time of 5 min and the solution was subjected to shaking at 230 rpm during accumulation.

In previous research of the authors the feasibility of using of nanogold for reagent-free preconcentration of Hg traces from waters has been shown [22]. In a three-step mechanism, the catalytic activity of nanogold leads to transformation of naturally occurring Hg species (Hg^2+^, CH_3_Hg^+^, (CH_3_)_2_Hg, PhHg^+^, C_2_H_5_Hg^+^ were tested) into elemental mercury (Hg^0^) which is then trapped by amalgamation. The ability of the gold-mesoporous silica films—in the following referred to as “sampling sticks”—to accumulate dissolved mercury (Hg^2+^) from a sample solution was tested in several measurement series. Beside concentration-dependent measurement, comparability of individually prepared batches of sampling sticks was investigated. Moreover, time-dependent accumulation and variation of active gold-coated area of sampling sticks were studied. Finally, recyclability of the sampling sticks was evaluated in order to estimate lifetime of the gold-mesoporous silica films with regard to Hg accumulation ability. First, concentration-dependent accumulation of Hg onto the gold-mesoporous silica films (1.5 cm^2^) was studied by immersing the sampling stick for 5 min in an aqueous solution containing a known concentration of dissolved HgCl_2_ in the ng·L^−1^ level. To quantify the amount of accumulated mercury on the sampling stick Hg^0^ was thermally desorbed and measured by AFS. For this purpose, the sticks were first rinsed and then inserted into a heating chamber coupled to the AFS. After drying the sampling stick with an argon gas stream the collector tube is heated to 550 °C. Thermally desorbed mercury from the sampling stick is then transported by an argon gas stream to a gold net collector within the AFS instrumentation in order to remove interfering water vapor from the gas stream, then it is thermally desorbed again and measured at a wavelength of 253.7 nm. As can be seen in Figure 4 the amount of Hg found on the sampling stick increases linearly with increasing concentrations of Hg in the sample solution confirming the feasibility of the sticks for Hg sampling and subsequent quantification. Moreover, the data obtained from a sampling stick without gold-coating (blind stick) shows almost no Hg accumulation (see Figure 4, data set D) proving that accumulation is driven quasi-exclusively by amalgamation of Hg with gold nanoparticles providing high selectivity. Accordingly, adsorption efficiency deriving from the slope of the linear regressions is 19 times higher for the sampling sticks compared to the blind stick. 

Furthermore, repetition of this experiment using films from different batches in terms of both, gold sputtering and mesoporous silica film deposition gave comparable results (see Figure 4). All film preparation iterations showed a linear response with virtually identical slopes, and only slightly varying absolute mercury read-outs in the mercury concentration range 5–25 ng·L^−1^. These results indicate a good batch-to-batch reproducibility of sampling stick preparation as well as Hg accumulation and release 

In order to estimate achievable detection limit under the above-given conditions, the sampling sticks were evaluated under even more dilute conditions (1–13 ng·L^−1^). Calculation on basis of the obtained calibration function according to Hubaux and Vos [29] resulted in of limit of detection (LOD) as low as 0.753 ng Hg L^−1^.

**Figure 5:**
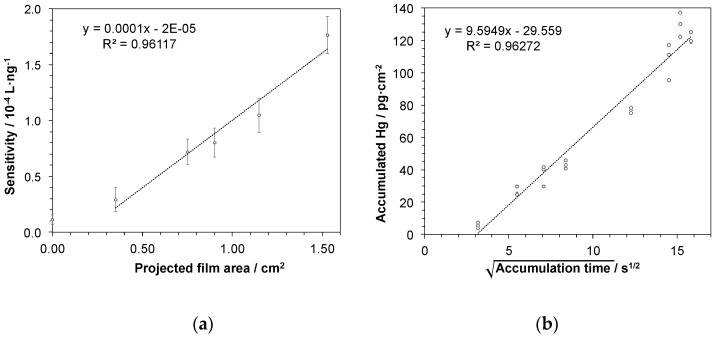
Sensitivity of the procedure for Hg determination depending on (**a**) the size of gold-coated area of sampling stick: Presented as slopes of the linear regressions deriving from accumulation experiments in a concentration range of 5–25 ng·L^−1^ Hg. Error bars represent uncertainty of slopes as derived from confidence interval of the linear regression with *N* = 15 and *P* = 95%; and (**b**) accumulation time of mercury onto the sampling stick: The square root of time-dependency suggests a diffusion-controlled accumulation. The mercury detection was performed by atomic fluorescence spectrometry. The concentration of the aqueous Hg^2+^ test solution was 100 ng·L^−1^ in a sample volume of 6 mL and the active gold area of the sampling stick was 1.5 cm^2^.

In a next series of experiments, Hg accumulation depending on the size of the active gold-coated area of the sampling stick was investigated. Hence, sampling sticks having gold areas ranging from about 0.3 cm^2^ to 1.5 cm^2^ were prepared and accumulation experiments as described above were performed. Here, it is to be expected that the capacity of Hg accumulation and thus the sensitivity of the procedure for Hg determination increases with larger gold area size. Therefore, linear regressions of the data obtained from concentration-dependent measurements for each stick were calculated providing slopes as measure for sensitivity. In Figure 5a the slopes are plotted against the gold area revealing a linear correlation between active gold area and sensitivity of Hg determination. Hence, sensitivity and thereby limit of detection of the proposed approach can be tuned by adapting the size of the sampling stick.

In order to study the kinetics of the mercury accumulation into the gold-mesoporous silica films, another set of measurements was performed at a fixed mercury concentration of 100 ng·L^−1^ varying accumulation times in a broad range from 10 s to 4.2 min. As shown in Figure 5b, a linear response was obtained when the amount of recovered mercury was plotted against the square root of the accumulation time, suggesting that the accumulation process is diffusion controlled within the investigated time range. On a related note, variation of the accumulation time adds yet another possibility to influence the sensitivity of Hg determination by this approach. Moreover, even as short accumulation times as 10 s may be enough for detecting even low levels of mercury, since the experiment here gives significantly higher value (8.5 ± 2.5 ng Hg) compared to blank value (2.4 ± 0.4 ng Hg). In addition, short accumulation times may be preferred in cases when the mercury concentration is high. In conclusion, considering that the film area easily can be adjusted, with the adaption of accumulation time, Hg trace determination at any given target concentration window should be possible.

**Figure 6:**
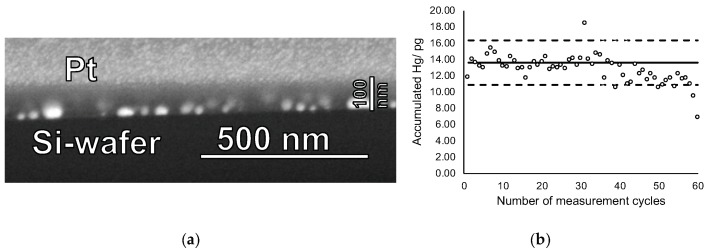
(**a**) FIB-SEM cross-section image of a gold nanoparticle-mesoporous silica film after 60 mercury accumulation-thermal release cycles and (**b**) accumulated amount of mercury on this sampling stick for the 60 cycles. The mercury concentration in the solution was 10 ng·L^−1^, the accumulation time 5 min, and the heat-treatment duration was 60 s at 550 °C. In (**b**) the solid line shows the expected amount of accumulated mercury as derived from the calibration function of sampling stick. Dashed lines represent variation range as derived from the residual standard deviation (SD) of the calibration function with a coverage factor k = 2.

Finally, cycling experiments were performed using mercury accumulation times of 5 min at a concentration of 10 ng Hg L^−1^ and heating cycles of 60 s. Even after 60 cycles no visual change of film structure nor changes in the film thickness were observed, suggesting that the films indeed can be used repeatedly without structural degradation (see Figure 6a). Figure 6b reveals that about 40 accumulation and measurement cycles can be performed before a slow decrease in accumulation efficiency occurred. However, significant loss of Hg accumulation efficiency was observed only after 60 cycles, most probably caused by to partial pore blocking due to repeated accumulation/heat-treatment cycling. Hence, lifetime of the new sampling stick is approximately 40–60 measurement cycles.

### 3.3. Analytical Performance and Validation of Hg Trace Analysis

In order to estimate achievable detection limit under the above-given conditions, the sampling sticks were evaluated under even more dilute conditions (1–13 ng·L^−1^). Calculation on basis of the obtained calibration function according to Hubaux and Vos [29] resulted in of limit of detection (LOD) as low as 0.753 ng Hg L^−1^ and a limit of quantification (LOQ) of 1.51 ng Hg L^−1^. These values reveal the feasibility of the proposed approach to investigate pristine natural waters where mercury concentrations in the low ng per litre range are to be expected. Moreover, the working range was found to be linear up to at least 100 ng Hg L^−1^ and could be extended to higher or lower concentrations, respectively, by fitting the active sampling area of the sticks (A_active_) and/or the accumulation time (t_acc_) as described in chapter 3.2 and Figure 5. Hence, the above given limits—working range, LOD and LOQ—are determined with the selected procedural parameters and subject to adaption depending on the requirement of the planned analysis. For instance, detection of Hg concentrations above 100 ng L^−1^ is achieved with a mere dipping of the stick into the samples for 10 s only, as was described in Section 3.2.

Precision was evaluated considering analytical repeatability as well as reproducibility in terms of usage of sampling sticks coming from different synthesis batches. Reproducibility in terms of batch-to-batch comparison of sampling sticks ranges between 5.1% and 13.8% (n ≥ 9) when calculating relative standard deviations (RSDs) for all data obtained at a given concentration obtained from different sampling sticks (see Figure 4). On the other hand, analytical precision, i.e., replicate Hg quantification using one and the same sampling stick for pre-concentration, is much higher with RSDs between 2.20 and 2.75% (see Table 1). In conclusion, these experiments confirm the feasibility of the proposed approach in ultra-trace analysis, where standard deviations for analyte concentration below 1 µg L^−1^ are typically in the range of 20–25% [30,31]. Most importantly, accuracy of the proposed method was proofed by successful recovery of Hg traces in a certified reference material (CRM), namely the river water ORMS-5. The Hg concentration found by application of two individual sampling sticks was 24.1 ± 2.6 ng Hg L^−1^ (stick A) and 24.1 ± 2.5 ng Hg L^−1^ (stick B) respectively. Both values do not significantly differ from the certified value of 26.2 ± 1.3 ng Hg L^−1^ (stick A: Δc = 2.07 ng Hg L^−1^ < U_c_ = 5.36 ng Hg L^−1^; stick B: Δc = 2.07 ng Hg L^−1^ < U_c_ = 5.18 ng Hg L^−1^; calculated according to *Linsinger* [32]). Table 1 summarizes all analytical figures of merit for the proposed approach as well as the data for validation by measurement of CRM.

## 4. Conclusions

Here, we have demonstrated that a novel design of sampling sticks based on supported gold nanoparticles covered by a thin mesoporous silica film allows for highly reproducible determination of trace levels of mercury in aqueous samples by atomic fluorescence spectrometry. The active part of the sampling sticks, gold nanoparticles, are first formed on the silicon substrate through thermal treatment of thin homogeneous gold films, after which the gold nanoparticles are coated with a thin layer of mesoporous silica. The mesoporous silica film serves to stabilize the gold nanoparticles as a layer preventing removal of the gold nanoparticles from the substrate under wet conditions and hindering further sintering of the gold upon thermal treatment. Thereby still full accessibility of the gold nanoparticles is ensured and accumulation of mercury follows a square root of time-dependency, suggesting a diffusion-controlled process. Moreover, high reproducibility in structural properties, gold loadings, as well as Hg accumulation efficiency is achieved with this approach. A linear response regarding Hg accumulation for the tested trace concentration range from 1 to 25 ng·L^−1^ is given with a high level of reproducibility (RDS: 5–14%). As to be expected, accumulation efficiency and with it sensitivity of the Hg determination scaled directly with the active film area provided for accumulation. Thus, the parameters sampling time and stick size can be tuned to fit a wide range of environmentally relevant mercury concentration levels. For an accumulation time of 5 minutes and a size of 1.5 cm^2^ a limit of detection as low as 0.753 ng·L^−1^ was achieved. This is fully in line with even the toughest upcoming regulations regarding mercury contents in aqueous media. Importantly, the films were recyclable for at least 30 cycles, often even 60 cycles, which makes these sampling sticks highly promising for environmental monitoring of mercury levels in waters through on-site sampling and detection. Future studies must be focused on the analysis of complex real-life samples, like natural saline and freshwaters, using these novel sampling sticks. Furthermore, the mesoporous silica film could be exchanged for other functional metal oxides, for example, TiO_2_, which is known to be able to oxidize organics upon exposure to (UV)-light, and which could exhibit higher hydrolytic stability. 

## Figures and Tables

**Table 1:** Analytical figures of merit and recovery in certified reference material. (Sampling parameters: *V* = 6 mL; t_acc_ = 5 min; A_active_ = 1.5 cm^2^; *n* = 4; *P* = 95%).

Parameters	Found Values
Linear working range ^1^	1–100 ng Hg L^−1^
Regression coefficient R^2^	0.9816
Precision given as RSD	
c(Hg) = 1 ng Hg L^−1^	2.39%
c(Hg) = 5 ng Hg L^−1^	2.75%
c(Hg) = 10 ng Hg L^−1^	2.20%
c(Hg) = 25 ng Hg L^−1^	2.45%
Accuracy given as recovery in CRM	92 ± 11%
ORMS-5: c(Hg)_cert._ = 26.2 ± 1.3 ng Hg L^−1^	
Sampling stick A ^2^	24.1 ± 2.6 ng Hg L^−1^
Sampling stick B ^2^	24.1 ± 2.5 ng Hg L^−1^
Sensitivity	
Limit of detection ^3^	0.753 ng Hg L^−1^
Limit of quantification ^3^	1.51 ng Hg L^−1^
Recyclability of sampling stick	≥30 cycles

^1^ Higher concentration was not tested in order not to contaminate the analytical set-up for trace analysis. ^2^ Given as mean ± uncertainty with *n* = 4. ^3^ Calculated on basis of the obtained calibration function according to Hubaux and Vos [29].

## Supplementary Materials

The following are available online at http://www.mdpi.com/2079-4991/9/1/35/s1. Figure S1. Calibration curve for CV-AFS measurement of Hg in a concentration range from 1 to 30 ng Hg L-1 with a sample volume of 2.2 mL corresponding to absolute Hg masses of 2.09 pg to 62.70 pg.
