# Supplementary material for: Mesoporous Silica-gold Films for Straightforward, Highly Reproducible Monitoring of Mercury Traces in Water
Source: Nanomaterials (Basel). 2018 Dec 28;9(1):35. doi: 10.3390/nano9010035 (PMC6359669; doi:10.3390/nano9010035)
Supplement: Supplementary file 1 [file nanomaterials-09-00035-s001.pdf]

Supporting Information for

Mesoporous silica-gold films for straightforward, highly reproducible monitoring of mercury traces in water

Anna Mutschler, Vivian Stock, Lena Ebert, Emma Björk, Kerstin Leopold* and Mika Lindén*

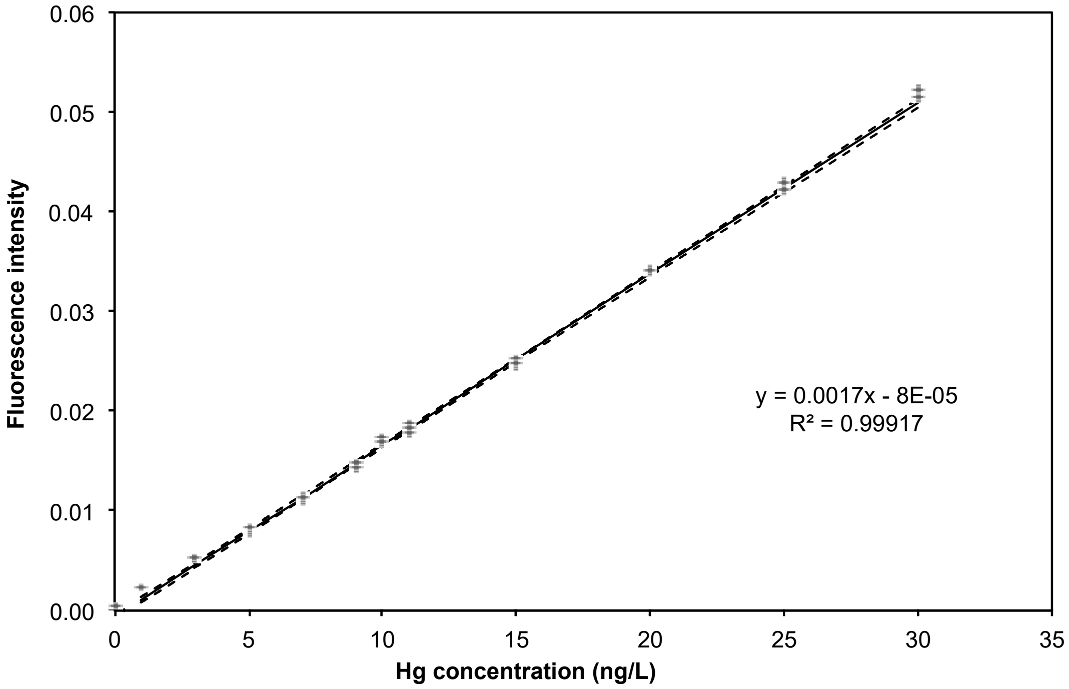

Figure S1. Calibration curve for CV-AFS measurement of Hg in a concentration range from 1 to 30 ng Hg L^{-1} with a sample volume of 2.2 mL corresponding to absolute Hg masses of 2.09 pg to 62.70 pg.
